# Brain Processes While Struggling With Evidence Accumulation During Facial Emotion Recognition: An ERP Study

**DOI:** 10.3389/fnhum.2020.00340

**Published:** 2020-09-03

**Authors:** Yu-Fang Yang, Eric Brunet-Gouet, Mariana Burca, Emmanuel K. Kalunga, Michel-Ange Amorim

**Affiliations:** ^1^CIAMS, Université Paris-Saclay, Orsay, France; ^2^CIAMS, Université d’Orléans, Orléans, France; ^3^Centre Hospitalier de Versailles, Hôpital Mignot, Le Chesnay, France; ^4^CESP, DevPsy, Université Paris-Saclay, UVSQ, Inserm, Villejuif, France; ^5^UVSQ, LISV, Université Paris-Saclay, Velizy, France

**Keywords:** P100, N170, P250, physiognomic features, diffusion decision model, emotional facial expression

## Abstract

The human brain is tuned to recognize emotional facial expressions in faces having a natural upright orientation. The relative contributions of featural, configural, and holistic processing to decision-making are as yet poorly understood. This study used a diffusion decision model (DDM) of decision-making to investigate the contribution of early face-sensitive processes to emotion recognition from physiognomic features (the eyes, nose, and mouth) by determining how experimental conditions tapping those processes affect early face-sensitive neuroelectric reflections (P100, N170, and P250) of processes determining evidence accumulation at the behavioral level. We first examined the effects of both stimulus orientation (upright vs. inverted) and stimulus type (photographs vs. sketches) on behavior and neuroelectric components (amplitude and latency). Then, we explored the sources of variance common to the experimental effects on event-related potentials (ERPs) and the DDM parameters. Several results suggest that the N170 indicates core visual processing for emotion recognition decision-making: (a) the additive effect of stimulus inversion and impoverishment on N170 latency; and (b) multivariate analysis suggesting that N170 neuroelectric activity must be increased to counteract the detrimental effects of face inversion on drift rate and of stimulus impoverishment on the stimulus encoding component of non-decision times. Overall, our results show that emotion recognition is still possible even with degraded stimulation, but at a neurocognitive cost, reflecting the extent to which our brain struggles to accumulate sensory evidence of a given emotion. Accordingly, we theorize that: (a) the P100 neural generator would provide a holistic frame of reference to the face percept through categorical encoding; (b) the N170 neural generator would maintain the structural cohesiveness of the subtle configural variations in facial expressions across our experimental manipulations through coordinate encoding of the facial features; and (c) building on the previous configural processing, the neurons generating the P250 would be responsible for a normalization process adapting to the facial features to match the stimulus to internal representations of emotional expressions.

## Introduction

The ability to recognize and distinguish between emotions conveyed by facial expressions is crucial for social cognition (Adolphs, 2002; Smith et al., 2005). The human brain is able to detect facial micro-expressions lasting about 500 ms (Yan et al., 2013), and even from a brief subliminal sample visual input (Vukusic et al., 2017). Within this time window, several visual pathways based on featural, configural, or holistic information cooperate to process faces (Tanaka and Gordon, 2011). Featural processing refers to the extraction of individual parts of the face, such as the eyes, nose, and mouth. These physiognomic features are crucial for emotion recognition (Scheller et al., 2012; Wegrzyn et al., 2017). Configural processing considers the spatial distances and relative positioning of local facial features, whereas holistic processing focuses on the integration of several features into a “Gestalt” or “all-in-one-piece” representation (Tanaka and Gordon, 2011; Piepers and Robbins, 2012). It is unclear how and when these different processes contribute to emotional face perception.

Investigating event-related potentials (ERPs) in electroencephalographic (EEG) signals is one of the most widely used methods for examining early face processing (Eimer and Holmes, 2002; Tanaka et al., 2006; Rossion and Caharel, 2011). Three major face-sensitive ERP components have been identified in the literature: the P100, N170, and P250. Although the P100 component is not always examined in face perception studies, it is sensitive to both bottom-up low-level features (e.g., color or contrast) and top-down attentional processes (Schweinberger, 2011). The P100 emerges on occipital channels at around 100 ms after face stimulus onset and exhibits larger responses to faces than to buildings and scrambled faces (Herrmann et al., 2005a), suggesting face sensitivity. Different facial expressions of emotion can be separated visually within 100 ms after stimulus onset (Liu and Ioannides, 2010). The face-sensitive N170 component, peaking at around 170 ms after stimulus onset, is maximal in occipital–temporal channels and is thought to reflect configural processing (Rossion et al., 1999; Bentin and Deouell, 2000; Eimer, 2000; Itier and Taylor, 2004). Although initially a matter of debate (Pessoa and Adolphs, 2010), there is growing evidence that subcortical regions like the amygdala are involved in differential responses to neutral vs. emotional facial expressions (especially threatening expressions) in both the P100 (Rotshtein et al., 2009; Méndez-Bértolo et al., 2016; Müller-Bardorff et al., 2018) and N170 (Conty et al., 2012). These results are consistent with the reciprocal modulatory effects between the amygdala and cortical regions involved in face processing (Luo et al., 2007; Garvert et al., 2014; Meaux and Vuilleumier, 2016; Sato et al., 2017). During face processing, the P250 emerges after the N170 over parietal sites between 200 and 300 ms post-stimulus and responds to emotional expression information (Eimer and Holmes, 2007; daSilva et al., 2016).

Studies have examined the modulation of the P100, N170, and P250 by manipulations tapping featural, configural, and holistic characteristics in order to understand the contribution of the corresponding processes during emotional facial recognition. While low-level features (e.g., color or contrast) and the configural relationship between facial features remain unchanged, turning faces upside down disrupts configural processing. This so-called face inversion effect (FIE) evokes larger and delayed P100 (Itier and Taylor, 2004) and N170 components (Itier and Taylor, 2004; Honda et al., 2007; Jacques and Rossion, 2007) than do upright faces. This modulation caused by the FIE suggests that both the P100 and N170 index an early stage of configural processing of facial features (Halit et al., 2000; Itier and Taylor, 2004; Herrmann et al., 2005b). In contrast, distorting configural information modulates the N170, but not the P100, suggesting that the N170 is particularly sensitive to the spatial processing of physiognomic features (Bentin and Deouell, 2000; Eimer, 2000).

Another way to investigate early face-sensitive processes is to degrade stimulus quality as much as possible while providing sufficient emotional information for the perceptual system to activate face-specific processing. Some studies have used this strategy to investigate brain response to sketched face stimuli. Sagiv and Bentin (2001) found that N170 latency and amplitude for upright schematic faces are similar to photographed faces, suggesting that “a face specific visual mechanism is triggered whenever a stimulus contains sufficient information to generate the concept of a face” (p. 942). In their experiment 1, the authors also investigated the N170 response to sketched faces. They found reduced amplitude and delayed latency for the N170 in response to faces sketched with richer details compared to simple schematic ones, supposedly due to the nature of their sketched face stimuli requiring more analytic visual processing. Indeed, the combination of the basic physiognomic features of sketched faces may be sufficient to evoke the formation of a face concept. Zhao et al. (2016) used composite faces (their experiment 3) to demonstrate that participants performed worse with misaligned photographed face stimuli than with correspondingly sketched rendered faces when they were instructed to judge whether the top halves of two sequentially presented faces were the same while ignoring the irrelevant bottom halves. These results suggest that, although sketched faces contain sufficient information to evoke the concept of a face, the removal of three-dimensional shape information from faces (brought by texture, shading, etc.) reduces holistic face processing.

Distorting the face stimulus also affects early face-sensitive ERPs. Burkhardt et al. (2010) showed that peak amplitude of the P250 decreased as the amount of perceived distortion of a compressed or expanded face increased. However, if the participants adapt to distorted faces, the P250 becomes increased again because the face appears more normal. In contrast, adaptation conditions that increase the perceived distortion of faces decreased the P250 amplitude. However, these authors only found small and inconsistent effects of adaptation on the N170, theorizing that “assuming that the neural generator of the P250 component lies downstream of the generator of the N170 component, these data imply that the neurons generating the P250 are adapting to facial features at the configural level” (p. 3755). The literature provides additional evidence suggesting that the P250 may reflect a normalization process in order to match the visual input to canonical (upright and typical) stored representations of faces (Marzi and Viggiano, 2007). The purpose of this normalization process is to compensate for transformations in the stimulus with respect to canonical representations by adjusting the percept (scale, orientation, etc.) in order to match it with stored representations (Ullman, 1989). In short, the P100, N170, and P250 appear to be relevant neuroelectric markers in order to investigate early-stage visual processes involved in the encoding of the emotional content of faces. Some authors even consider that they somehow assemble together in a “positive-negative-positive (P100-N170-P250) ERP complex” (Puce et al., 2013).

**Figure 1:**
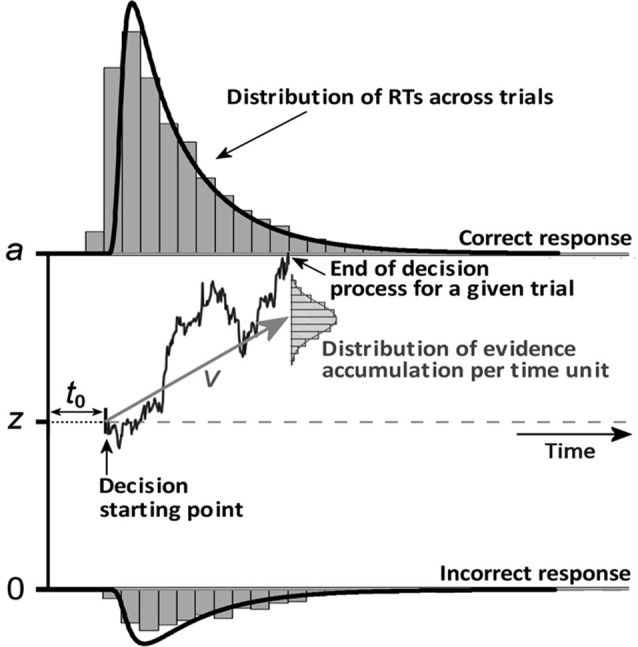
An illustration of several parameters of the diffusion decision model (DDM). Perceptual decision-making supposedly relies on the accumulation of noisy sensory evidence from an initial state (*z*) to a response threshold (*a*). A response is made when the level of sensory evidence exceeds the threshold of the response corresponding to a given percept. The initial evidence value at which accumulation starts is the starting point parameter (*z*), which reflects participants’ (*a priori*) response bias toward a given response. If the participant is unbiased, then *z* = *a*/2. The drift rate (*v*) at which the evidence accumulates in the diffusion process corresponds to the average distance traveled vertically per time unit according to a normal distribution. Finally, the non-decision time *t*_0_ is an additive constant in the reaction time (RT) that includes both stimulus encoding and the motor components (response preparation and execution).

Using the high temporal resolution of EEG, we were also able to investigate the chronometric characteristics of the neural response, which could be predictive of psychophysical performance. A growing literature links brain imaging with behavioral data using mathematical models of decision-making in forced-choice tasks (Philiastides et al., 2006; Ratcliff and McKoon, 2008). Sequential sampling models assume that decision-making is founded on samples of a stream of information from sensory signals. In the present study, we used the diffusion decision model (DDM; see Ratcliff and McKoon, 2008), illustrated in Figure 1. Two parameters were of particular interest in this study: non-decision time (*t*_0_) and drift rate (*v*). Non-decision time is an additive constant in the reaction time (RT) that includes both a perceptual (the time it takes to encode the stimulus) and a motor component (movement preparation and execution). Drift rate (the slope of evidence accumulation) reflects the quality of evidence accumulation from the sensory information provided by the stimulus. Evidence supposedly accumulates at a constant rate despite moment-by-moment noise of external (at stimulus level) or internal (its cognitive representation) origin. Easier-to-process stimuli lead to faster initial encoding (i.e., shorter non-decision time, *t*_0_) and faster evidence accumulation (i.e., greater drift rate). The study of Bushmakin and James (2013) showed that large inversion effects on faces were mediated by a slower rate of perceptual evidence accumulation. Previous EEG studies of visual recognition using single-trial analysis have demonstrated that both early (N170) and later (arising around 220 ms post-stimulus onset) components correlate with decision difficulty in a face vs. car categorization task (Philiastides et al., 2006).

### Purpose of the Study

The aim of the present study was to investigate the relationships between early face-sensitive ERP components and sensory evidence accumulation during emotion recognition. This perceptual categorization activity requires quick but complex analysis of local physiognomic features and of their spatial configuration on a face. To achieve this, several experimental factors were manipulated to impose constraints on perceptual systems and to elicit specific sources of variance: (i) quality was varied while providing sufficient emotional information with the use of sketched vs. photographed face stimuli (*Stimulus Type* factor); and (ii) configural processing was emphasized using the classical face inversion paradigm (*Orientation* factor). Several methodological choices characterize this study. First, unlike photographed face stimuli, the sketched face stimuli are stylized and appear under quite diverse forms across the literature, which do not always allow for strict condition matching or replication (e.g., Benson and Perrett, 1991; Leder, 1996; Meinhardt-Injac et al., 2013; Zhao et al., 2016). Thus, in order to compare conditions and to avoid potential confounding effects of stylized stimuli, the sketched face stimuli were computer-generated from validated photographed stimuli of the Radboud Faces Database (RaFD; Langner et al., 2010) Second, both the amplitudes and latencies of the P100-N170-P250 ERP complex were measured in order to be introduced into the analyses of the DDM variables.

The main goals of the study were: (1) to test whether both upright photographed and sketched faces provide similar behavioral performance outcomes, demonstrating that both stimulus sets are well matched and convey sufficient information to make an emotion recognition decision; (2) to demonstrate that photographs elicit the FIE as reflected in behavioral and ERP measures (P100, N170, and P250); (3) to replicate the findings by Sagiv and Bentin (2001) that the ERP FIE differs between sketches and photographs; (4) to verify that upright photographed faces match our internal representations of emotional expressions by testing that these easier-to-process stimuli lead to faster initial encoding (i.e., shorter non-decision time) and faster sensory evidence accumulation (i.e., greater drift rate); and (5) to study how P100-N170-P250 complex characteristics are related to decision processes as measured by DDM variables when specific sources of variance are induced by manipulating either the Orientation or Stimulus Type.

## Experiment

### Materials and Methods

#### Participants

Twenty-five healthy volunteers (six females and 19 males, mean age = 26.4 ± 6.5 years, range = 20–40 years) were recruited through advertisements on campus and at the Sport Sciences faculty of Université Paris-Saclay and reported normal or corrected-to-normal vision. Twenty subjects were right-handed according to a translated version of the Edinburgh Handedness Inventory (Oldfield, 1971). The present study involving human participants was reviewed and approved by EA 4532 institutional review board at Université Paris-Sud/Saclay. The participants provided written informed consent to participate in this study, including for publication of the results.

#### Stimulus Presentation

**Figure 2:**
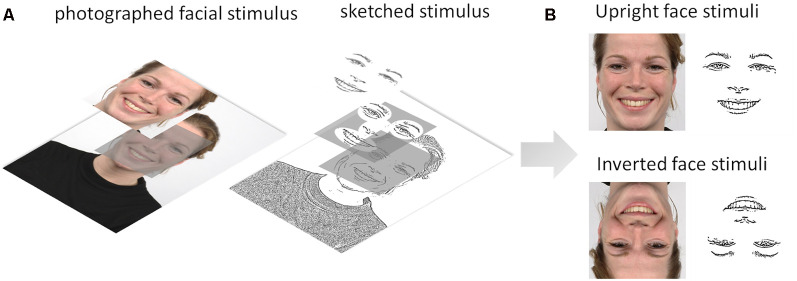
**(A)** Illustration of the image processing steps from the Radboud Faces Database (RaFD) photographed facial stimulus to sketched stimulus using the sketch filter in OpenCV. **(B)** Upright and inverted photographed and sketched stimuli for a happy facial expression.

Two sets of facial expression stimuli were used in the experiment: sketched facial stimuli and their photographed face counterparts chosen from the RaFD (Langner et al., 2010). We applied a sketch filter in OpenCV to the photographed facial stimuli to create a set of sketched stimuli retaining the eyes, eyebrows, nose, and mouth (physiognomic features) and excluding insignificant features such as hair, spots, et cetera (see Figure 2). These two subsets were validated and selected from previous behavioral and eye-tracking experiments on emotion recognition using reaction times and unbiased hit rate as the criteria and fixation measures (Yang, 2018). Each stimulus set contained five facial expressions: fearful, angry, sad, happy, and neutral. Each emotion category included five upright and five inverted facial stimuli, and gender identity was randomized and counterbalanced across the emotions. All of the images were cropped and resized to 400 × 400 pixels. The facial stimuli subtended a 10° × 10° visual angle and were presented centrally on a 17-inch computer monitor (resolution, 1,280 × 1,024 pixels) with a 60-Hz refresh rate. The value of the visual angle conformed to the one observed in normal face-to-face interaction (Henderson et al., 2005). We used E-prime 2.0 software to conduct the experiment.

#### Procedure

The task was forced-choice facial emotion recognition in which the participants were required to select the emotion displayed by a facial expression stimulus from five possible responses: neutrality, anger, fear, happiness, and sadness. The experiment consisted of two successive parts: (1) practice session (see Appendix A in Supplementary Material for details); and (2) experimental session.

The experimental session comprised two blocks (one for sketched faces and the other for photographed faces) of 150 trials each. The procedure was similar to the practice session, with participants pressing the key corresponding to the displayed emotion, except that no feedback on performance was provided. There were 50 stimuli per block: 10 expressors (five upright and five inverted) × five emotions, and each was repeated randomly three times within the blocks. Each block was separated by a pause, and the block order was counterbalanced across all participants. Each trial began with a 600-ms fixation cross, then a 300-ms blank screen (used as a baseline period for EEG analysis), followed by the facial stimulus displayed against a white background remaining on the screen until the participants responded. Although the response time was self-paced with no time limit, the participants were instructed to respond as quickly and accurately as possible and to avoid blinking while the stimulus was being displayed.

#### EEG Acquisition

EEG recordings were performed using the BrainAmp system with active 32 Ag–AgCl electrodes (ActiCap; Brain Products, Munich, Germany; map of all electrode positions: https://www.brainproducts.com/files/public/downloads/actiCAP-64-channel-Standard-2_1201.pdf). Electrode impedances were below 20 kΩ. The FCz electrode was used as a reference and AFz was used as a ground electrode. Vertical eye movements such as blinks were derived from the differential signal between Fp2 (on the EEG head cap) and Fp1, relabeled as vertical electrooculography (VEOG), positioned on the right infraorbital ridge, with both electrodes aligned vertically (see Sullivan, 1993). EEG signals were measured continuously at a sampling rate of 1,000 Hz and a 50-Hz notch filter was applied. BrainVision Analyzer was used to process the EEG data offline. The data were bandpass-filtered between 0.1 and 40 Hz (zero-phase shift Butterworth filter, 24 dB/octave). Nonspecific electrical artifacts were semi-automatically detected and then removed with a 200-ms margin from all channels except Fp2 and VEOG (criteria: differences between the maximal and minimal voltage superior to 100 μV, voltage steps superior to 50 μV/ms, and low electrical activity inferior to 0.5 μV in a 200-ms interval). Ocular artifacts were then processed using ocular correction with independent component analysis (ICA; Delorme et al., 2007). Infomax restricted ICA was performed on the whole data and channels except VEOG and Fp2. Between one and two independent components (ICs, mean = 1.5) were rejected (set to zero) across participants, on average. One IC was rejected among 14 participants, two ICs among 10 participants, and three ICs for one participant. The data were then segmented into 1,100-ms epochs, applying a baseline correction (100 ms before stimulus onset and 1,000 ms interval after stimulus onset). Those epochs were checked again for nonspecific abnormalities potentially induced by successive corrections (adding the following criterion to the above-described criteria: absolute amplitudes above 200 μV were rejected as artifacts). Last, they were averaged for each condition and participant. Note that only correct response trials were used to investigate the EEG signals concerning behavioral performance.

### Data Analysis

#### Behavior

Accuracy was calculated as an unbiased hit rate (*Hu*) for each emotional state category to control for potential response biases (Biehl et al., 1997; Goeleven et al., 2008; Langner et al., 2010). Indeed, accuracy is usually estimated by the proportion of correctly identified target face stimuli (i.e., simple hit rate) and does not consider participant’s response biases (e.g., false alarms, et cetera). The *H*_u_ for each emotion category was calculated on a choice matrix with targeted and chosen expressions as rows and columns, respectively. Then, the number of responses in each cell were squared and divided by the product of the marginal values of the corresponding row and column. *H*_u_ varies between 0 and 1 (1 = correctly identified target) and has to be arcsine transformed before analysis (Wagner, 1993).

Instead of running statistical analysis on RTs *per se*, we computed variables of interest resulting from a DDM analysis of RTs, as described in “Introduction” section. These DDM parameters were computed on RTs for correct and incorrect answers using *Fast-dm* software (Voss et al., 2004; Voss and Voss, 2007) after excluding RTs greater than mean + 3 SD on an intra-individual basis for each condition separately, along the same lines as other authors aiming to investigate the electrophysiological correlates of decision-making with diffusion models (e.g., Mueller et al., 2017). DDM is typically applied to two alternative decision tasks, whereas in our case we used a five (emotional expressions) forced-choice task. However, DDM can be used on multiple-choice data if no decision bias criterion is met (Voss et al., 2013). *Fast-dm* estimates *z* with *z*_r_ = *z*/*a* (relative starting point) scaled from 0 to 1, with *z*_r_ = *a*/2 = 0.5 indicating the absence of a decisional bias. Therefore, we let “*z*_r_” be a free parameter (Voss and Voss, 2007) in order to verify that our data complied with the no decision bias requirement in the Stimulus Type (photographs vs. sketches) × Orientation (upright vs. inverted) conditions. Statistical analysis of the DDM variable *z*_r_ showed that diffusion modeling could be applied to our data since none of our four conditions of interest differed significantly from 0.5 (see “Behavioral Data” section). Finally, the diffusion model fitting of our data was excellent, with all *p*s > 0.95, across the four conditions (N.B.: significant *p*-values are indicative of model misfit; see Voss and Voss, 2007). Further analysis of the quality of individual DDM fits is available in Supplementary Material, Appendix B.

*H*_u_, RTs, and diffusion model variables were then compared across Stimulus Type and Orientation as within-subject factors using repeated-measures ANOVAs. Bonferroni correction *post hoc* comparisons were used to examine significant effects of interest involving more than two means. However, unless otherwise stated, if the *Stimulus Type* × *Orientation* interaction was significant, we only report the impoverishment effect for each level of the Orientation factor as well as the inversion effect for each level of Stimulus Type. We also provide 95% confidence intervals and Cohen’s *d* values as measures of effect size.

#### Electrophysiology

As pre-processing, we first excluded trials with RTs >3,357 ms from the ERP analysis. This value corresponded to 3 SD (3 × 1,119 ms) of the entire RT dataset, i.e., approximately mean RT (1,171 ms) + 1.6 SD, leading to the exclusion of 5.3% of the correctly answered trials. With the incorrectly answered trials also excluded from the ERP analysis, this yielded an average of 63 out of 75 trials per condition (range = 53–67) across 25 participants. The epochs were averaged separately for each Stimulus Type (photographs and sketches) and Orientation (upright and inverted). Three clear visual ERP components were identified and analyzed: the P100, N170, and P250. Peak latencies of the P100, N170, and P250 were extracted at their maximum absolute amplitudes within different time windows: 100–180 ms for the P100, 163–240 ms for the N170, and 217–280 ms for the P250. The peak amplitudes of P100, N170, and P250 were quantified as the maximum absolute voltage amplitude whether in terms of positive values at the P100 and P250 or negative values at the N170 time window. Different pairs of electrodes were analyzed depending on the ERP of interest: the P100 at occipital electrodes (O1 and O2), the N170 at occipito-temporal electrodes (P7 and P8), and the P250 at parietal electrodes (P3 and P4). These electrode sites have been used previously in the face perception literature for the P100 (Herrmann et al., 2005a,b), the N170 (Itier et al., 2006; Eimer, 2011; Rossion and Caharel, 2011), and the P250 (Feng et al., 2009). Along the lines of Sagiv and Bentin (2001), who used experimental manipulations similar to ours, we examined the N170 only at P7/P8 channels. The peak amplitude and latency of the P100, N170, and P250 were analyzed separately using repeated-measures ANOVAs with Stimulus Type (photographs and sketches), Orientation (upright and inverted), and *Hemisphere* (left and right) as the within-subject factors. Bonferroni-corrected *post hoc* pairwise comparisons were followed up for significant effects involving more than two means and Cohen’s *d* estimated the effect sizes. The data were analyzed using Brain Analyzer (Brain Products, Munich, Germany) and JASP software (version 0.11.1). We set the reference significance threshold at 0.05 for both the behavioral and EEG data analyses.

#### Multivariate Analyses

Of the different more or less sophisticated methods employed to link EEG with behavioral data in decision-making (O’Connell et al., 2012; Kelly and O’Connell, 2013; Bridwell et al., 2018), we chose to adopt a multivariate approach to investigate the common sources of variance shared by those variables (e.g., Schubert et al., 2015). We first computed the inversion effect (mean values for the inverted faces minus mean values for the upright faces) and the Stimulus Type effect (mean values for the sketch faces minus mean values for the photo faces) for each participant and each variable of interest. Then, we conducted a correlation analysis followed by a principal component analysis (PCA) if the Kaiser–Meyer–Olkin (KMO) measure of sampling adequacy was greater than 0.50 and Bartlett’s test for sphericity was significant. Correlation matrices were computed using both Pearson’s *r* coefficient and Spearman’s ρ coefficient (the *r* value of the rank-transformed data). Reporting Spearman’s correlation coefficients has two advantages, namely: ρ is less affected by outliers and it does not make the assumption (contrary to the Pearson’s *r*) that intervals on the different variable scales measure the same psychological/neurocognitive unit (see Howitt and Cramer, 2017, p. 102). Indeed, we cannot assume that a change in one unit of drift rate (*v* scale) is equivalent to a change in one unit of an ERP variable. For the sake of simplicity, the correlation matrices (together with bilateral significance) are provided in the Supplementary Material, so here, we will focus on the PCA results when the above-mentioned criteria were met. PCAs were performed on the Pearson’s *r* correlation matrix, followed by a varimax rotation. Each PCA included five variables of interest: *v*, *t*_0_, P100, N170, and P250. The observations were the mean values of each of the 25 participants for the inversion effect in each stimulus type condition (resulting in 50 values per variable of interest) and for the impoverishment effect in each stimulus orientation condition (*idem*). Due to the small number of observations, we ran separate PCAs to examine the link between ERPs and DDM variables, depending on the effect under study and whether it concerned ERPs peak amplitude or peak latency. Along the lines of Jolliffe (2002), we retained factorial solutions that accounted for at least 70% of cumulative variance, with an eigenvalue cut-off at 0.80 rather than 1 (Jolliffe, 2002, pp. 113–115). Loadings greater than 0.50 (i.e., when a factor accounts for more than 25% of the variable variance) will be highlighted.

## Results

### Behavioral Data

**Figure 3:**
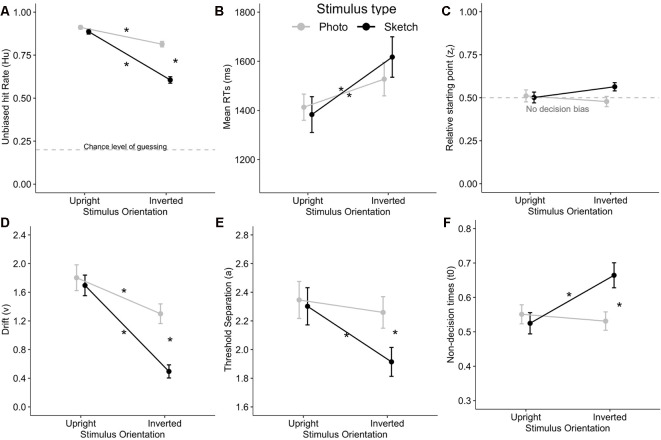
Stimulus Type × Orientation interaction on unbiased response accuracy *H*_u_ **(A)**, mean correct answer reaction times (RTs; **B**), as well as on the diffusion decision model (DDM) variables such as relative starting point (*z*_r_) with non-decision bias at 0.5 **(C)**, drift (*v*) **(D)**, threshold separation (*a*; **E**), and the non-decision time (*t*_0_, in seconds; **F**). Stars indicate Bonferroni-corrected *p* < 0.05. Error bars indicate the standard error of the mean.

Although the *H*_u_ values were arcsine transformed before running the ANOVA as required (Wagner, 1993), we provide untransformed mean values in the text and figures for the sake of readability (because when *H*_u_ > 0.85, arcsine-transformed values will exceed 1). The ANOVA revealed significantly greater accuracy (*H*_u_) for the photographed facial stimuli than for the sketched faces (*F*_(1,24)_ = 75.24, *p* < 0.001, $\eta_{\text{p}}^{2}$ = 0.76). *H*_u_ deteriorated with stimulus inversion (*F*_(1,24)_ = 80.85, *p* < 0.001, $\eta_{\text{p}}^{2}$ = 0.77). Stimulus Type × Orientation interaction was significant (*F*_(1,24)_ = 28.82, *p* < 0.001, $\eta_{\text{p}}^{2}$ = 0.55). *Post hoc* analysis indicated that the accuracy for upright faces did not differ significantly (*p* = 1). The stimulus inversion effect was found for both the photographed (*p* < 0.001, Cohen’s *d* = 0.88) and sketched faces (*p* < 0.001, *d* = 2.09), although the inversion effect affected more sketched faces (*M*_effect_ = 0.28) than photographs (*M*_effect_ = 0.10; see Figure 3A).

The ANOVA showed significantly greater RTs in response to the inverted facial stimuli than for upright faces (*F*_(1,24)_ = 67.22, *p* < 0.001, $\eta_{\text{p}}^{2}$ = 0.74). However, the mean RTs did not differ between the photographed and sketched stimuli (*F*_(1,24)_ < 1, n.s.), nor did the Stimulus Type × Orientation interaction reach significance (*F*_(1,24)_ = 3.00, *p* > 0.09, $\eta_{\text{p}}^{2}$ = 0.11; see Figure 3B).

As a prerequisite for DDM analysis, we examined the *z*_r_ (relative starting point) parameter in order to verify that our data complied with the no decision bias requirement corresponding to *z*_r_ = *a*/*2* = 0.5 (see “Behavior” section) in each cell of the Stimulus Type (photographs vs. sketches) × Orientation (upright vs. inverted) conditions. The ANOVA on *z*_r_ showed no significant main effect or any interaction for the two factors (all *p*s > 0.05). Moreover, Bonferroni-corrected *post hoc* tests (*p*s > 0.05/4) showed that diffusion modeling could be applied to our data since none of our four conditions of interest significantly differed from 0.5 (see Figure 3C).

The ANOVA on drift (*v*) showed better performance for the photographed than for the sketched faces (*F*_(1,24)_ = 24.97, *p* < 0.001, $\eta_{\text{p}}^{2}$ = 0.51) and for the upright than for the inverted stimuli (*F*_(1,24)_ = 50.71, *p* < 0.001, $\eta_{\text{p}}^{2}$ = 0.68). In addition, there was a significant Stimulus Type × Orientation interaction (*F*_(1,24)_ = 9.29, *p* = 0.006, $\eta_{\text{p}}^{2}$ = 0.28). *Post hoc* analysis indicated a decrease in *v* with the inversion effect for both photographed (*p* = 0.02, *d* = 0.61) and sketched faces (*p* < 0.001, *d* = 1.45), together with a greater decrease for inverted sketched as compared to inverted photographed faces (*p* < 0.001, *d* = 1.10). Interestingly, this interaction is very consistent with the interaction pattern on *H*_u_ (see Figures 3A,D).

The ANOVA on the threshold separation showed that more information is processed for photo faces compared to sketched faces (*F*_(1,24)_ = 5.28, *p* = 0.03, $\eta_{\text{p}}^{2}$ = 0.18) and for upright faces with respect to inverted faces (*F*_(1,24)_ = 7.21, *p* = 0.01, $\eta_{\text{p}}^{2}$ = 0.23). A significant Stimulus Type × Orientation interaction was observed (*F*_(1,24)_ = 4.42, *p* < 0.05, $\eta_{\text{p}}^{2}$ = 0.16). Subsequent *post hoc* analysis revealed that a significantly smaller amount of information was processed for inverted sketches compared to each other condition (all *p*s < 0.02; see Figure 3E).

The ANOVA on non-decision time (*t*_0_) indicated that *t*_0_ did not significantly differ between photos and sketches (*F*_(1,24)_ = 2.67, *p* = 0.12, $\eta_{\text{p}}^{2}$ = 0.1). However, non-decision times were significantly longer for inverted faces than for upright faces (*F*_(1,24)_ = 5.26, *p* = 0.03, $\eta_{\text{p}}^{2}$ = 0.18). Furthermore, there was a significant Stimulus Type × Orientation interaction (*F*_(1,24)_ = 16.60, *p* < 0.001, $\eta_{\text{p}}^{2}$ = 0.41). *Post hoc* analysis revealed that *t*_0_ was significantly greater (by about 120 ms) for inverted sketched faces compared to upright sketched faces (*p* < 0.001) and to inverted photo faces (*p* = 0.007) and more marginally compared to upright photos (*p* = 0.057; see Figure 3F).

### ERP Results

#### P100 Component

**Figure 4:**
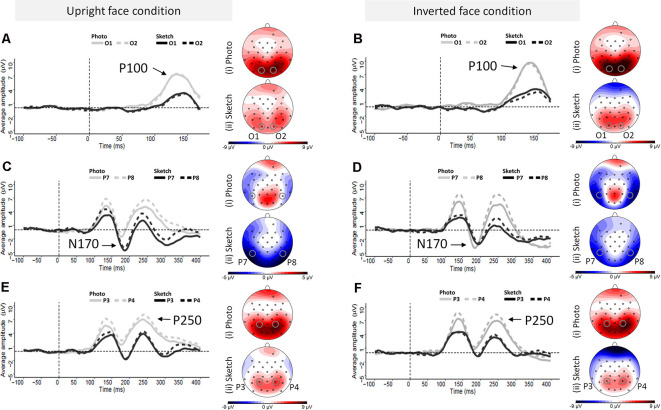
Grand average event-related potential (ERP) waveforms elicited by photographed and sketched faces in the upright (*left column*) and inverted (*right column*) orientations at the left and right occipital electrodes (*O1* and *O2*, respectively) for the P100 component **(A,B)**, the left and right occipito-temporal electrode sites (*P7* and *P8*) for the N170 component **(C,D)**, and the left and right parietal electrode sites (*P3* and *P4*) and the left and right at parietal electrodes (*P3* and *P4*) for P250 component **(E,F)**.

**Figure 5:**
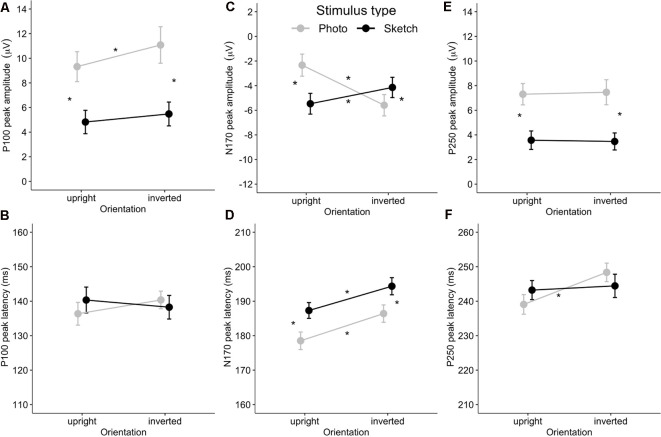
Mean event-related potential (ERP) peak latency and amplitude for the Stimulus Type × Orientation interaction for the P100 **(A,B)**, N170 **(C,D)**, and P250 **(E,F)**. Error bars indicate standard error of the mean. Stars indicate Bonferroni-corrected *p* < 0.05.

As illustrated in Figures 4A,B, the amplitudes of P100 were larger for the photographed face stimuli (in gray) than for the sketched face stimuli (in black) over occipital channels. The ANOVA showed greater P100 amplitude in response to the photographed faces compared to the sketched faces (*F*_(1,24)_ = 73.81, *p* < 0.001, $\eta_{\text{p}}^{2}$ = 0.76) and in response to the inverted stimuli compared to the upright stimuli (*F*_(1,24)_ = 11.05, *p* = 0.003, $\eta_{\text{p}}^{2}$ = 0.32; see Figure 5B). There was a non-significant Stimulus Type × Orientation interaction (*F*_(1,24)_ = 2.35, *p* = 0.14, $\eta_{\text{p}}^{2}$ = 0.09), with a parallelism pattern suggestive of an additive effect of Stimulus Type and Orientation on the P100 amplitude (see Figure 5A). It is interesting to note that the size of the impoverishment effect (95% CI = 3.84–6.26 μV, *d* = 1.72) was much greater (in terms of mean effect) and robust (as measured by Cohen’s *d*) than the moderate inversion effect (95% CI = 0.46–1.96 μV, *d* = 0.66). Finally, there was no effect of Hemisphere on the P100 amplitude, nor any other significant interaction between factors (all *p* > 0.21).

The ANOVA on P100 peak latency showed no main effects (all *p*s > 0.45), nor any significant interaction between the experimental factors (all *p*s > 0.065; see Figure 5B).

#### N170 Component

Visual examination of the grand average ERP waveforms indicates larger N170 components for the sketched faces in the upright condition (see Figure 4C) over occipito-temporal electrodes. The ANOVA showed greater N170 amplitude in response to inverted faces than for the upright stimuli (*F*_(1,24)_ = 9.08, *p* < 0.01, $\eta_{\text{p}}^{2}$ = 0.27). The only other significant effect was the Stimulus Type × Orientation interaction (*F*_(1,24)_ = 67.73, *p* < 0.001, $\eta_{\text{p}}^{2}$ = 0.74; see Figure 5C). *Post hoc* analyses of the sketched stimuli revealed greater amplitude for the upright stimuli compared to the inverted stimuli (*p* = 0.019). In contrast, for the photographed stimuli, the amplitude was greater for the inverted than for the upright stimuli (*p* < 0.001; see Figure 5D). The effect size of the absolute inversion effect for the sketched faces (95% CI = 0.15–2.49 μV, *d* = 0.62) was moderate compared to the photographed faces (95% CI = 2.09–4.42 μV, *d* = 1.53), for which the inversion effect was greater and more robust. Finally, *post hoc* comparisons showed smaller negativity for the upright photographed faces compared to the upright sketched and inverted photographed faces (all *p*s < 0.04).

The ANOVA showed greater N170 peak latency for the sketches than for the photographs (*F*_(1,24)_ = 19.32, *p* < 0.001, $\eta_{\text{p}}^{2}$ = 0.45) and for the inverted stimuli than for the upright stimuli (*F*_(1,24)_ = 42.85, *p* < 0.001, $\eta_{\text{p}}^{2}$ = 0.65). The non-significant Stimulus Type × Orientation interaction (*F*_(1,24)_ < 1, n.s.) suggests an additive effect of Stimulus Type (8 ms increase for the sketched faces) and Orientation (7 ms increase for the inverted facial stimuli) on the N170 peak latency (see Figure 5D). The effect sizes of the inversion effect (95% CI = 5–10 ms, *d* = 1.31) and of the Stimulus Type effect (95% CI = 5–12 ms, *d* = 0.89) were of comparable magnitude. Finally, there was no main effect of Hemisphere, nor did Hemisphere interact with the other factors (all *p*s > 0.09).

#### P250 Component

As illustrated in Figures 5E,F, the P250 waveforms were greater over the parieto-occipital region for the photographed face stimuli than for the sketched face stimuli in both the upright and inverted conditions. Likewise, the ANOVA showed only significantly greater P250 amplitude in response to the photos compared to the sketched faces (*F*_(1,24)_ = 79.63, *p* < 0.001, $\eta_{\text{p}}^{2}$ = 0.77). No other main effect or interaction was observed (all *p*s > 0.11).

The ANOVA on the P250 peak latency showed a significant increase for the inverted faces compared to the upright faces (*F*_(1,24)_ = 6.4, *p* = 0.02, $\eta_{\text{p}}^{2}$ = 0.21). There was no other significant main effect (all *p*s > 0.65). However, the effect of Orientation varied significantly with Stimulus Type (*F*_(1,24)_ = 4.51, *p* = 0.04, $\eta_{\text{p}}^{2}$ = 0.16), providing additional information. *Post hoc* analyses showed a significant inversion effect for the photos (*p* = 0.01, *d* = 0.66), but not for the sketched faces (*p* = 1). The only other significant effect was an interaction between Hemisphere and Stimulus Type (*F*_(1,24)_ = 5.85, *p* = 0.02, $\eta_{\text{p}}^{2}$ = 0.20). However, *post hoc* analysis revealed no significant pairwise comparisons (all *p*s > 0.74).

### Multivariate Analyses Results

A first analysis was conducted on the inversion effect with the DDM and ERP amplitude variables on the data for the photographed (*n* = 25) and sketched (*n* = 25) stimuli together. The characteristics of the Pearson’s *r* matrix (KMO = 0.52, Bartlett’s test *p* = 0.02) were used to conduct a PCA, summarized in Table 1, after varimax rotation. Factor 1 accounted mainly for the inversion effect on *t*_0_ and N170 amplitude, sharing respectively 76% (*t*_0_) and 50% (N170) of their variance with factor 1. The positive correlation between both variables (Pearson’s *r* = 0.33, *p* = 0.018; Spearman’s *ρ* = 0.37, *p* = 0.009) reflects the fact that the FIE on *t*_0_ decreases with increasing N170 deflection in response to FIE (see Supplementary Figure S4). In contrast, the additional variance explained by factor 2 accounted mainly for the inversion effect on the P100 and P250. In addition, the inversion effect on the P100 amplitude was shared between factors 2 and 3, the latter reflecting a common source of variance of the inversion effect with *v*. This is consistent with the positive (although marginal) correlation (see Supplementary Table S2) between both variables, whereby the greater the face inversion on the P100 amplitude the less the evidence accumulation (*v*) will be impaired (see Supplementary Figure S5).

**Table 1:** Principal component analysis (PCA) solution on the inversion effects after a varimax rotation.

	Factor number			Communalities
	1	2	3	*h*^2^
*v*	−0.24	−0.01	**0.85**	0.77
*t*_0_	**0.87**	−0.21	0.02	0.80
P100 peak amplitude	0.16	**0.61**	**0.59**	0.75
N170 peak amplitude	**0.71**	0.19	−0.30	0.63
P250 peak amplitude	−0.10	**0.92**	−0.03	0.85

*Factor 1 reflected the fact that the FIE on t_0_ decreased with increasing N170 deflection in response to FIE (see also Supplementary Figure S4). Factor2 accounted mainly for the inversion effect on the P100 and P250. In addition, the FIE on the P100 amplitude was shared between factors 2 and 3. Factor 3 reflected the fact that the greater the FIE on the P100 amplitude the less the evidence accumulation (v) was impaired (see also Supplementary Table S2)*.

The following analysis was conducted on the inversion effect on both the DDM and ERP latency variables. The characteristics of the correlation matrices (KMO = 0.48, Bartlett’s test *p* = 0.43) precluded further investigation using PCA. Apart from a significant negative correlation between the inversion effects on *v* and *t*_0_ (Spearman’s *ρ* = −0.287, *p* = 0.044; illustrated in Supplementary Figure S6), there was a significant positive correlation between the inversion effects on the N170 and P250 latencies (Pearson’s *r* = 0.298, *p* = 0.036; Spearman’s *ρ* = 0.290, *p* = 0.041; see Supplementary Table S3). The former effect reflects the fact that the more the inversion effect degrades drift (the “*v* for inverted faces” minus “*v* for upright faces” difference becomes more negative), the more it will increase *t*_0_ (the “*t*_0_ for inverted faces” minus “*t*_0_ for upright faces” difference becomes more positive). Similarly, the positive correlation between the inversion effects on ERPs suggests that the greater the inversion effect on the N170 latency, the greater the inversion effect on the P250 latency. According to Vovk–Sellke maximum *p* ratio (VS-MPR; see Sellke et al., 2001), the odds in favor of these correlations over *H*_0_ were greater than 2.68.

Another analysis was conducted on the *impoverishment* effect on the DDM and ERP amplitude variables on the data for the upright (*n* = 25) and inverted (*n* = 25) stimuli together. The characteristics of Pearson’s *r* matrix (KMO = 0.53, Bartlett’s test *p* = 0.04) were used to conduct a PCA, summarized in Table 2. Factor 1 accounted mainly for the Stimulus Type effect on P100 and P250, sharing 69% (P100) and 64% (P250), respectively, with factor 1. Factor 2 reflected a common source of variance for the Stimulus Type effect on *v* and on the N170 amplitude. This is consistent with the negative correlation (about −0.27, VS-MPR > 2.19; see Supplementary Table S4) between both variables, whereby stimulus impoverishment tends to increase the N170 negative deflection and degrade evidence accumulation. Finally, factor 3 reflected the Stimulus Type effect on *t*_0_ amplitude including a slightly shared variance with *v*.

**Table 2:** PCA solution on the impoverishment effects after a varimax rotation.

	Factor number			Communalities
	1	2	3	*h*^2^
*v*	0.07	**−0.60**	−0.47	0.58
*t*_0_	0.05	0.06	**0.94**	0.89
P100 peak amplitude	**0.83**	−0.28	−0.06	0.78
N170 peak amplitude	0.04	**0.87**	−0.01	0.76
P250 peak amplitude	**0.80**	0.31	0.10	0.75

*Factor 1 accounted mainly for the Stimulus Type effect on both P100 and P250 peak amplitude. Factor 2 reflected the fact that stimulus impoverishment tends to increase the N170 deflection and to degrade evidence accumulation (see also Supplementary Table S4). Finally, factor 3 reflected mainly the Stimulus Type effect on t_0_*.

The next analysis was conducted on the *impoverishment* effect on the DDM and ERP latency variables. The characteristics of the Pearson’s *r* matrix (KMO = 0.51, Bartlett’s test *p* = 0.003) were used to conduct a PCA, summarized in Table 3. Factor 1 accounted mainly for the Stimulus Type effect on the N170 and P250 latencies. In addition, the effect of Stimulus Type on the DDM variables fell under factor 2, with which none of the ERP latencies shared noticeable variance. Moreover, the effect on the P100 latency was explained by a separate source of variance (factor 3).

**Table 3:** PCA solution on the impoverishment effects after a varimax rotation.

	Factor number			Communalities
	1	2	3	*h*^2^
*v*	−0.01	**0.74**	0.29	0.63
*t*_0_	−0.11	**−0.83**	0.15	0.73
P100 peak latency	0.05	0.06	**0.96**	0.93
N170 peak latency	**0.90**	−0.08	−0.01	0.82
P250 peak latency	**0.86**	0.21	0.08	0.80

*Factor 1 accounted mainly for the Stimulus Type effect on the N170 and P250 latencies. In addition, the effect of Stimulus Type on the diffusion decision model (DDM) variables fell under factor 2, with which none of the event-related potential (ERP) latencies shared noticeable variance. Finally, factor 3 accounted for the impoversihment effect on the P100 latency in a separate source of variance*.

## Discussion

We studied how neural responses (particularly the P100, N170, and P250 neurocognitive markers) and decision-making parameters (from DDM) varied when local and configural information of the faces was manipulated by means of the stimulus type (photographs vs. sketches) and orientation (upright vs. inverted) during an emotion recognition task. Despite the established importance of physiognomic features in the processing of upright and inverted faces, the essential relationship between the neural correlates of early visual processing and behavioral performance during emotional face recognition remains relatively unexplored. Therefore, we used a multivariate approach (correlation analyses possibly followed by PCA) to further explore the common sources of variance between the experimental effects on ERPs and the DDM parameters.

Upright photographs induced close-to-optimal performance, as reflected by a high level of accuracy (*H*_u_) and fast drift rate (*v*) with short non-decision time (*t*_0_), together with shorter N170 latency and decreased N170 amplitude, compared to the other conditions. Modifying stimulus quality by a change in orientation (FIE) or with sketched faces (impoverishment effect) had different neurocognitive consequences. Face inversion undoubtedly degraded the performance at the behavioral level, although emotion recognition remained well above the chance level. Moreover, the impact of the FIE on performance was even greater for impoverished stimuli (sketched faces). More precisely, not only was the drift rate degraded but non-decision times also increased in parallel, as documented by the effect of our experimental manipulations, both in terms of mean values and multivariate analysis. Assuming that the motor component of *t*_0_ (i.e., response preparation and motor response) was constant across conditions, we may reasonably interpret that the detrimental effect on *t*_0_ of combining stimulus inversion and impoverishment reflects an increase in the stimulus encoding component of *t*_0_ in RTs. Interestingly, our experimental manipulations, which aimed to challenge configural processing of emotional faces, had different consequences on the ERP and DDM parameters, suggesting distinct functional roles for each component of the P100-N170-P250 complex.

Face processing literature suggests that the P100 reflects an initial holistic gist of a face (Tanaka and Xu, 2018), supposedly providing a rough initial frame of reference (i.e., eyes-above-nose-above-mouth) to interpret the stimulus. It facilitates the identification of the physiognomic features that are processed subsequently at the configural level (as indexed by the N170). The much greater P100 amplitude in response to photographed faces (as compared to sketched faces) reflects the default adaptive tuning of its underlying neural generator to close-to-natural stimuli. Accordingly, the increased P100 amplitude due to face inversion may reflect a bottom-up attentional effect triggered by subcortical regions such as the pulvinar (Ward et al., 2007; Nguyen et al., 2013) in response to a natural face in an atypical orientation. The fact that the FIE on drift rate (*v*) and on the P100 amplitude share common variance (the greater the P100 amplitude, the less evidence accumulation will be impaired; see Supplementary Figure S5) suggests that the P100 neural generator provides a crucial support to the decision-making process in order to overcome the challenge posed by face inversion. This support may correspond to a holistic frame of reference specifying the natural eyes-above-nose-above-mouth face structure, whereas configural processing analyses in greater detail the relational and distance metrics between the physiognomic features. This distinction would correspond to the difference between the categorical (first-order relations) and coordinate (second-order relations) encoding of spatial relations in visual cognition (Kosslyn et al., 1992; Maurer et al., 2002), respectively.

N170 is a neuroelectric component indexing facial structure encoding at the crossroads of our experimental manipulations tapping configural processing of facial expressions. Indeed, the mean additive effects of stimulus inversion and stimulus impoverishment on the N170 latency, together with the fact that each condition except upright faces increased the N170 amplitude, are key findings along those lines. Furthermore, the FIE on non-decision times decreased with FIE increasing N170 amplitude (see Supplementary Figure S4). Assuming that the variation in non-decision times reflects the variation in stimulus encoding times, this suggests that the activity of the neural generator of the N170 must be increased in order to counteract the FIE. This is consistent with Sagiv and Bentin (2001), suggesting difficulty in encoding face structural content during emotion recognition. Since upright and inverted emotional photographed faces essentially share the same physical signals, the inversion effect is more likely to reflect a modulation of both the holistic (Itier and Taylor, 2002; Herrmann et al., 2005a; Joyce and Rossion, 2005) and configural processes (Piepers and Robbins, 2012), as indexed by the P100 and N170, respectively. Here, we theorize that the purpose of the underlying neural activity would be to maintain the structural cohesiveness of the facial expression (N170 neural generator), building on the initial holistic frame of reference of the face (P100 neural generator). Conjointly, this structural binding process may contribute to extract information on the subtle configural changes associated with different facial emotions. However, if key elements are missing, such as the head contour in our sketch stimuli, encoding the face within a holistic frame of reference will be hampered, thereby dramatically increasing *t*_0_ and reducing the rate of accumulation of evidence. Indeed, external features such as the hair and chin are represented holistically (Andrews et al., 2010) and thereby facilitate the localization of internal features such as the eyes and mouth. Furthermore, face inversion disrupts the categorical relations of the facial features (e.g., the mouth situated above eyes) within a viewer-centered frame of reference, while the coordinate relations remain unchanged (Niebauer, 2001). Consequently, as coordinate encoding relies on categorical information, fine-grained configural processing would equally be impaired (Maurer et al., 2002).

In order to decide which emotional expression is displayed despite our experimental manipulations, a normalization process is needed to match the visual input to typical upright representations of faces stored in long-term memory. Several findings are consistent with the hypothesis that the neurons generating the P250 adapt to the facial features processed at the configural level by the neural generator of the N170 (Burkhardt et al., 2010). On the one hand, FIE on the N170 and P250 latencies shared common variance (about 9%) that was even much greater (>25%) regarding the effect of stimulus impoverishment on these ERP latencies. Assuming that mental representations of facial expressions are stored in close-to-natural format, these results suggest that the matching process indexed by the P250 was much more challenging when applied to stimuli such as sketches (atypical faces) than when applying the normalization process to inverted stimuli (to match them to our upright mental representations of typical faces). On the other hand, FIE on the P250 dramatically increased the mean peak latency in response to photographed faces, whereas FIE had no effect on the P250 latency to sketched faces that remained at an intermediate level. The latter result suggests a more feature-based matching process for sketched faces. Furthermore, our data provide evidence of a shared variance between the P100 and P250 amplitudes regarding face inversion and stimulus impoverishment effects, which may suggest that the holistic frame of reference supported by the neural generator of the P100 contributes to the normalization process indexed by the P250.

Regarding the effect of stimulus impoverishment, the different behavioral parameters (whether from *H*_u_ or the DDM analysis of RT) showed no performance difference between upright face stimuli. This suggests that the physiognomic features of our sketched faces supplied sufficient expressive information for emotion recognition and that additional visual information provided by photographed faces (e.g., hair, face contour, wrinkles, etc.) may be less crucial for the task. However, this equivalent level of performance came at a neurocognitive cost. For example, neuroelectric correlates showed delayed latency and increased N170 amplitude for the upright sketched faces in comparison to the upright photographed faces, suggesting an increased difficulty in decoding the structural aspect of configural information from sketched faces. A recent behavioral study showed that participants do not perform as well for photographed faces compared to sketched faces in a face identification task when the top and bottom halves of a face were not aligned (i.e., a gap between them, known as the composite effect; Zhao et al., 2016). These results support the importance of configural processing of the structural integrity of the face as in upright photographed faces. This integrity is disrupted in sketched faces, leading to enhanced processing of featural information (i.e., eyes, nose, and mouth), which can be advantageous when the presentation is distorted, for example by misaligning facial parts. In the present study, this processing of physiognomic features for sketched faces was realized through the prism of configural processing, as indexed by the additive effect of our experimental manipulations on the N170 latency as well as by the increased encoding subcomponent of the non-decision times for inverted sketches. When compared to the results for upright faces, our upright sketch data seem consistent with the fact that the rate of sensory evidence accumulation is positively associated with the efficacy of information processing, which depends on the strength of the sensory information (Ratcliff, 1978). Similarly, in a face vs. car categorization task using single-trial EEG analysis of stimuli varying in visual noise, Philiastides et al. (2006) showed that the drift rate is greater for face images containing more sensory information than for the degraded face stimuli.

One interpretation of these findings is that the poorer the bottom-up feed-forward stimulus information is, the more the brain needs to exert top-down recurrent feedback in order to interpret the sensory input. Depending on the stimulus quality (whether in terms of details or orientation difference with respect to the canonical upright orientation), the brain would engage different face representations (either stored face templates or rule-based information) of given emotions that compete for the final decision (Palmeri and Cottrell, 2010; Palmeri et al., 2015). Both featural and holistic processing would run in parallel in response to the visual input. The P100 would reflect initial bottom-up holistic processing to activate relevant representations in order to understand the stimulus content (either a photographed or sketched face). In our experiment, prototypes of facial expressions close to the depicted facial expressions (e.g., anger and disgust) would be instantiated. However, when the stimulus quality is rich and sufficiently close to the representation characteristics (e.g., upright close-to-natural faces), sensory evidence quickly accumulates in favor of a given emotion. In contrast, if the stimulus is degraded (e.g., sketched faces with no head contour), additional recurrent processing is required to support the configural processing of the emotional content (conveyed by physiognomic features) of the face (*via* the N170 neural generator) and match the stimulus to stored representations (*via* the P250 neural generator). If necessary, rule-based processing of face content (e.g., in order to check emotional compatibility between the upper and lower face content) would assist the processing of the physiognomic content and facilitate decision-making. This additional processing is supposedly subsequent to the activity underlying the P100-N170-P250 complex. Certainly, the latter ERP complex is but a small piece of the big picture. Although it corresponds to just 25% of the neurocognitive processing time ending with the observable response from overt behavior, its contribution to the decision-making process is crucial, as supported by our findings. Still, in view of the remaining 75% of neurocognitive processing time that remains to be explored, the amount of variance in the decision-making process explained by this ERP complex is certainly appreciable.

The present study suffers from several limitations. First, our DDM analysis assumed that our experimental effects of interest (FIE and stimulus impoverishment) would not vary with the emotion displayed in the face stimuli. This was not completely the case, as detailed in Supplementary Material, Appendix D. However, although the response times and accuracy varied with the different facial expressions, the proportion of variance for the Stimulus Type × Orientation interaction was two to three times greater than for the higher-order interaction after including the *Emotion* factor. The fact remains that our trial number per facial emotion per participant and per condition was not high enough to properly study the relation between ERPs and the DDM variables for each emotion separately. Another related shortcoming is our participant sample size that limited our multivariate analysis. The PCA factorial solutions, retained on the basis of standard criteria (in terms of accounted cumulative variance and eigenvalue cutoff; see Jolliffe, 2002), indicated a link between several ERPs (P100 and N170) and two DDM variables (*t*_0_ and *v*). However, the underlying correlations mainly supported a link between N170 and the DDM variables. Nevertheless, a much larger sample size would be necessary to achieve stable estimates for the correlations that we evidenced (Schönbrodt and Perugini, 2013). Despite this, we were able to provide consistent and complementary findings across our different statistical approaches, which only future replication will be able to confirm or refute.

The aim of our study was to understand better the contribution of the P100-N170-P250 complex to decision-making when recognizing the emotional content of facial expressions. This ERP complex supposedly indexes early-stage visual processes involved in the encoding of the emotional content of faces. The mean duration of the non-decision times corresponding to our upright stimuli (about 525 ms) leading to the greatest recognition accuracy clearly supported this hypothesis. We may reasonably consider that the motor component of non-decision times did not vary across conditions and was constant at about 300 ms (minimum intercept value of choice RTs as a function set size (see Teichner and Krebs, 1974; Luce, 1991). Accordingly, the duration of the P100-N170-P250 complex fits well within the duration of the stimulus encoding component of the non-decision times. The fact that our experimental effects on the N170, non-decision time, and drift rate shared common variance points to the crucial role of this ERP complex for emotion recognition decision-making. In short, our study is original because it investigated the effect of experimental manipulations challenging these processes within the framework of DDM. Twenty years after the publication of the seminal article by Perrett et al. (1998) demonstrating that the rate of accumulation of neuronal activity increases with the departure of the faces from the canonical upright orientation, “accumulation of evidence” appears to be a relevant concept to understand the functional links between neuroelectric activity and decision-making.

## Conclusion and Perspectives

To our knowledge, our findings are the first to suggest that physiognomic features provided by sketched faces may convey sufficient information for emotion recognition, but at the expense of a neurocognitive adaptation during which the brain struggles to accumulate sensory evidence in favor of a given emotion. Although this conclusion holds because our stimuli were selected to be as unambiguous as possible, even upright photographed faces of so-called basic emotions (Ekman and Friesen, 1976) can be ambiguous [e.g., disgust may be confused with anger, fear with surprise; see Susskind et al. (2007)]. Therefore, the context in which the facial expression is incorporated (such as body posture; see Aviezer et al., 2008) proves crucial for interpreting the physiognomic features. Interestingly, the ERP components later than the ones we examined are sensitive to the compatibility between the facial expression and a situational context, such as the late positive potential (LPP; see Dozolme et al., 2018 for the effect of sentences preceding the faces) and the N400 (see Calbi et al., 2017 for the effect of body posture on the facial emotion). The role of these components in emotion recognition decision-making should be investigated further in order to test and extend the model we have proposed. This opens exciting avenues for future research investigating the neurofunctional reorganization of both early and late processes in populations suffering from a social cognition deficit, such as schizophrenia or autism, known for their atypical face processing (see Watson, 2013).

## Data Availability Statement

All datasets generated for this study are included in the article/Supplementary Material.

## Ethics Statement

The study involving human participants was reviewed and approved by EA 4532 Institutional review board at Université Paris-Sud/Saclay. The participants provided their written informed consent to participate in this study, including for publication of the results.

## Author Contributions

Y-FY conceived and designed the experiment, performed the experiments, collected and analyzed the data, prepared figures, authored or reviewed drafts of the article, and approved the final draft. EB-G conceived and designed the experiment, authored or reviewed drafts of the article, and approved the final draft. MB collected the data. EK collected the data. M-AA conceived and designed the experiment, analyzed the data, prepared figures, authored or reviewed drafts of the article, and approved the final draft.

## Conflict of Interest

The authors declare that the research was conducted in the absence of any commercial or financial relationships that could be construed as a potential conflict of interest.
