# Supplementary material for: Brain Processes While Struggling With Evidence Accumulation During Facial Emotion Recognition: An ERP Study
Source: Front Hum Neurosci. 2020 Sep 3;14:340. doi: 10.3389/fnhum.2020.00340 (PMC7497730; doi:10.3389/fnhum.2020.00340)
Supplement: Supplementary file 1 [file Data_Sheet_1.docx]

***Supplementary Material***

# Appendix A

## Practice Session of the experiment

The Practice Session included two sub-sessions: (1) a key learning session to ensure the participant was able to correctly associate a given response with a specific emotional state; (2) the facial emotion recognition session. The “D”, “F”, “H”, “J” and “K” keys of an AZERTY keyboard were re-labeled with stickers as follows: “C” (for “Colère”= anger in French); “P” (for “Peur” = fear); “N” (for “Neutre” = neutrality), “T” (for “Tristesse” = sadness), and “J” (for “Joie” = happiness). The key learning session consisted of a minimum of 30 trials with one emotional state word displayed randomly in the center of a screen to which participants responded by pressing the corresponding key. After each five-trial block, the average response accuracy was displayed (in %). This key learning session ended when participants reached 100% accuracy over five successive blocks, after, which the facial emotion recognition practice session began. There were two blocks, i) inverted and upright sketched faces; ii) inverted and upright photographed faces. Block order was randomized. After each block of five trials, the average response accuracy (in %) was displayed. The practice trials consisted of 40 images for the sketched and photographed face blocks: 2 Expressors x 2 Orientation (inverted vs. upright) x 2 Stimulus type (photographs vs. sketches) x 5 Facial expression. The stimuli used in the practice sessions were excluded from the experimental session. The participants obtaining less than 75% accuracy during the practice session did not take part in the experimental session.

# Appendix B

## Analysis of the individual Diffusion Decision Model fits

Predicted and empirical cumulative density functions (CDFs) of reaction times were computed for each participant separately, using *Fast-dm* (see Supplementary Figure 1A). In addition to the *p* values of model fitting provided by *Fast-dm*, we assessed statistically the overall quality of individual DDM model fits. Therefore, we computed the linear regression adjusted to the predicted CDFs values as a function of the empirical CDFs values for correct response RTs (see Supplementary Figure 1B). Table 1 summarizes the regression statistics that proved to be satisfactory with *R*² values greater than 0.98.


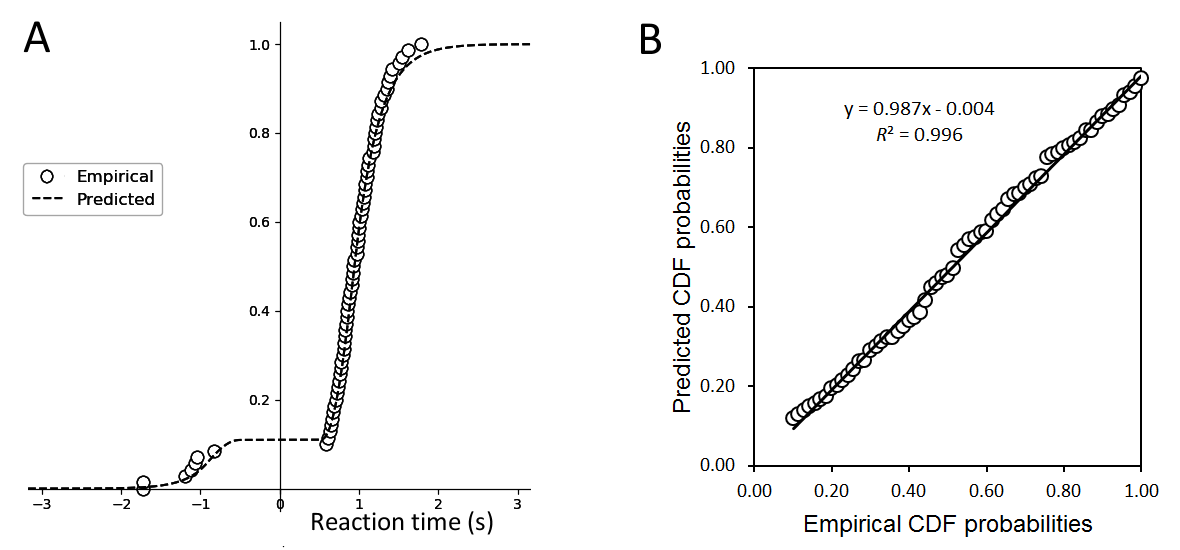


**Supplementary Figure 1.** Example of the data for one participant, in the inverted photographed faces condition. (A) *Fast-dm* plot of predicted and empirical CDFs as a function of RTs with negative signs assigned to error responses (Voss, Nagler, et al., 2013; Voss & Voss, 2007). (B) Linear fit adjusted to the predicted CDFs values plotted against the empirical CDFs values for correct response RTs.

| Predicted vs empirical CDFs regressions | Photographed faces | | Sketched faces | |
| --- | --- | --- | --- | --- |
|  | Upright | Inverted | Upright | Inverted |
| Mean individual *R*² | 0.9916 | 0.9910 | 0.9920 | 0.9873 |
| 95% CI | [0.9888; 0.9944] | [0.9886; 0.9934] | [0.9899; 0.9941] | [0.9849; 0.9898] |
|  |  |  |  |  |
| Mean slope | 0.9873 | 0.9872 | 0.9816 | 0.9687 |
| 95% CI | [0.9798; 0.9949] | [0.9770; 0.9973] | [0.9720; 0.9912] | [0.9566; 0.9808] |
| *p* values, slope vs 1 | 0.002 | 0.016 | 0.001 | 0.000 |
|  |  |  |  |  |
| Mean intercept | 0.0011 | -0.0007 | 0.0021 | 0.0100 |
| 95% CI | [-0.0054; 0.0076] | [-0.0059; 0.0046] | [-0.0044; 0.0087] | [0.0014; 0.0186] |
| *p*s, intercept vs 0 | 0.729 | 0.800 | 0.512 | 0.024 |

**Supplementary Table 1.** Summary of the linear regression statistics on the CDF values for correct response RTs.

Some predicted values were slightly underestimated with respect to the empirical values. This is evidenced by the mean slope values (of the predicted vs. empirical CDFs regressions) ranging between 0.95 and 1 (see 95% CI values in Table 1), and by a graphical analysis of model fit. *Supplementary Figure 2* and *3* show the mean predicted values (y-axis) of the CDF of the RT distributions plotted against mean empirical values (x-axis), along the same lines as (Voss, Rothermund, et al., 2013, Appendix B). Most of the data points fell on the unity line, suggesting that there was no systematic bias in the DDM fits.


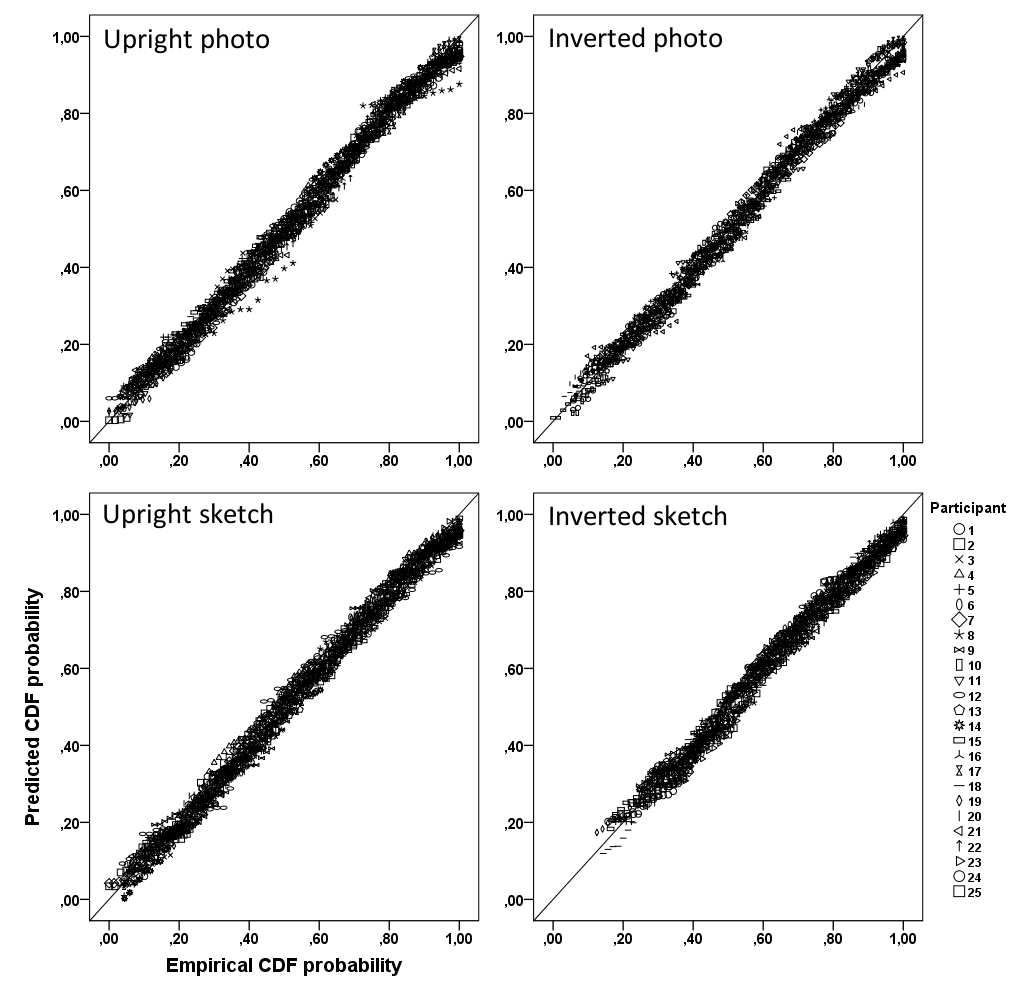


**Supplementary Figure 2**. Relation of the empirical vs predicted statistics (cumulative distribution function probability) of correct response RTs for each trial and experimental condition, across each participant. Each data point represents one trial.


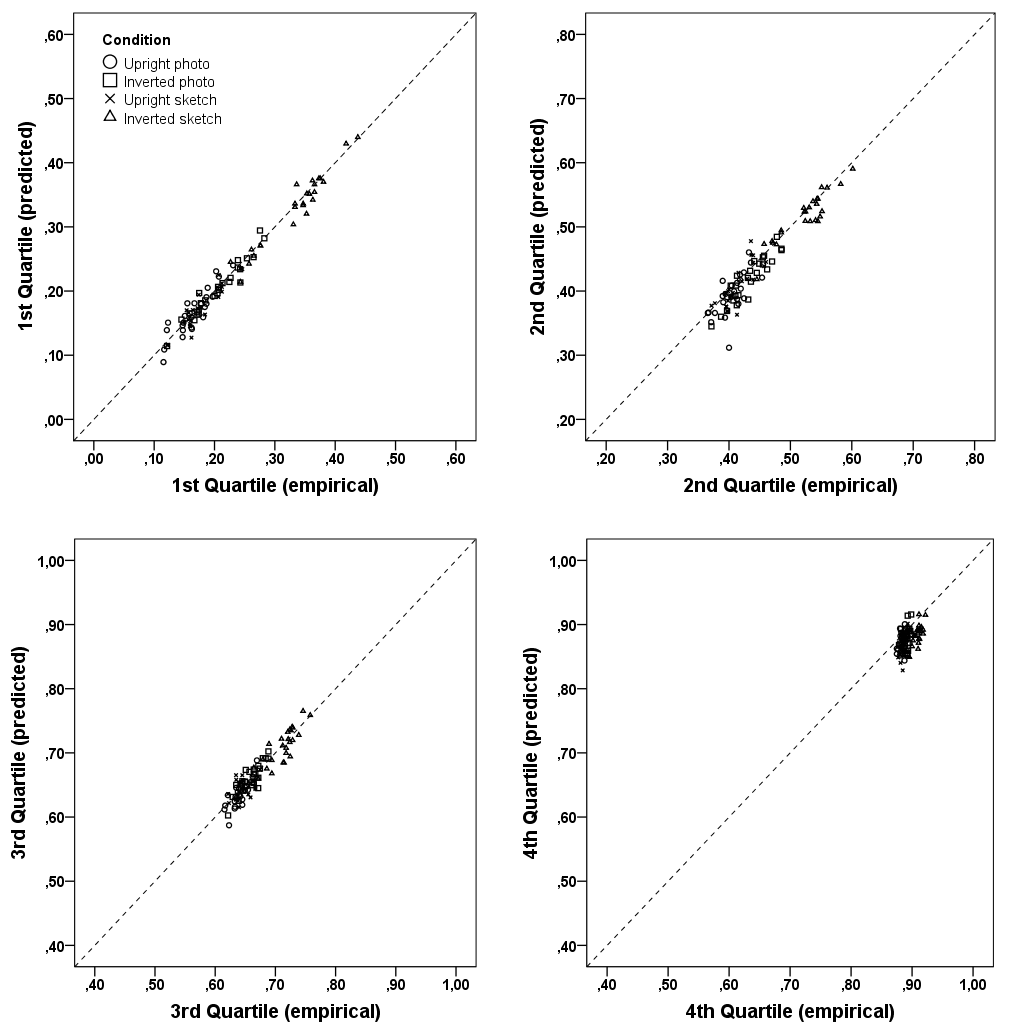


**Supplementary Figure 3**. Relation of the empirical vs predicted statistics (CDF probability) for each RT quartile of correct responses, across each experimental condition. Each data point represents the mean CDF probability of one participant in a given condition. Note that the more clustered data points for the 4^th^ Quartile reflect the characteristics of the CDFs (see *Supplementary Figure 1A*) where greater RT values keep increasing while probability values are capped close to 1.

# Appendix C

|  |  | |  | Pearson | | | | |  | Spearman | | | | |
| --- | --- | --- | --- | --- | --- | --- | --- | --- | --- | --- | --- | --- | --- | --- |
|  |  | |  | r | *p* | VS-MPR† | Lower 95% CI | Upper 95% CI |  | rho | *p* | VS-MPR† | Lower 95% CI | Upper 95% CI |
| *v* | - | *t*_0_ | | -0.242 | 0.091 | 1.691 | -0.487 | 0.039 |  | -0.287***** | 0.044 | 2.688 | -0.523 | -0.009 |
| *v* | - | P100_amp | | 0.242 | 0.090 | 1.694 | -0.039 | 0.488 |  | 0.235 | 0.101 | 1.592 | -0.047 | 0.482 |
| *v* | - | N170_amp | | -0.221 | 0.124 | 1.424 | -0.470 | 0.061 |  | -0.270 | 0.058 | 2.216 | -0.510 | 0.009 |
| *v* | - | P250_amp | | 0.082 | 0.569 | 1.000 | -0.200 | 0.353 |  | 0.044 | 0.760 | 1.000 | -0.237 | 0.319 |
| *t*_0_ | - | P100_amp | | -0.017 | 0.904 | 1.000 | -0.294 | 0.262 |  | -0.052 | 0.717 | 1.000 | -0.326 | 0.229 |
| *t*_0_ | - | N170_amp | | 0.332***** | 0.018 | 5.020 | 0.060 | 0.559 |  | 0.368****** | 0.009 | 8.757 | 0.100 | 0.586 |
| *t*_0_ | - | P250_amp | | -0.159 | 0.269 | 1.041 | -0.419 | 0.125 |  | -0.150 | 0.298 | 1.020 | -0.411 | 0.134 |
| P100_amp | - | N170_amp | | -0.048 | 0.740 | 1.000 | -0.322 | 0.233 |  | -0.127 | 0.379 | 1.000 | -0.391 | 0.157 |
| P100_amp | - | P250_amp | | 0.370****** | 0.008 | 9.349 | 0.102 | 0.588 |  | 0.338***** | 0.017 | 5.332 | 0.065 | 0.563 |
| N170_amp | - | P250_amp | | 0.043 | 0.766 | 1.000 | -0.238 | 0.318 |  | -0.049 | 0.736 | 1.000 | -0.323 | 0.233 |

**Supplementary Table 2.** Correlation matrices on the inversion effect for DDM parameters and ERPs peak amplitude. †Vovk-Sellke Maximum *p-*Ratio based on the *p* -value, the maximum possible odds in favor of H_1_ over H_0_ equals 1/(-e *p* log( *p* )) for *p* ≤ 0.37 (Sellke, Bayarri, & Berger, 2001). **p*< 0.05, ** *p* < 0.01, *** *p* < 0.001


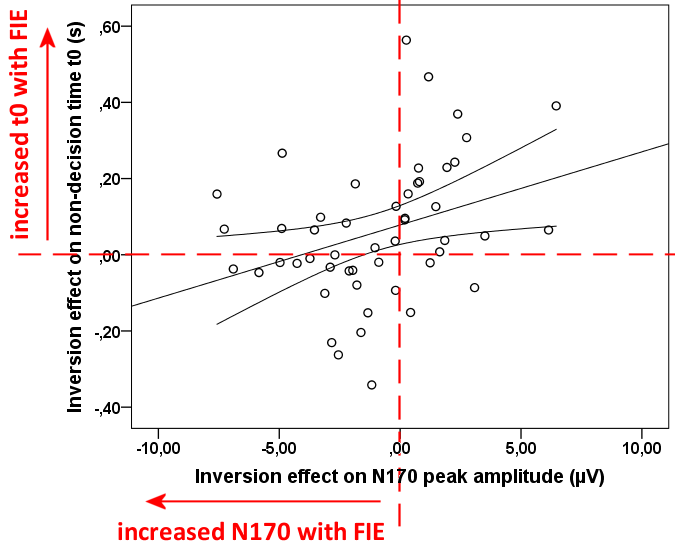


**Supplementary Figure 4.** Illustration of the positive link between FIE on *t*_0_ and on N170 peak amplitude. FIE on *t*_0_ decreases with increasing N170 deflection (in response to FIE).


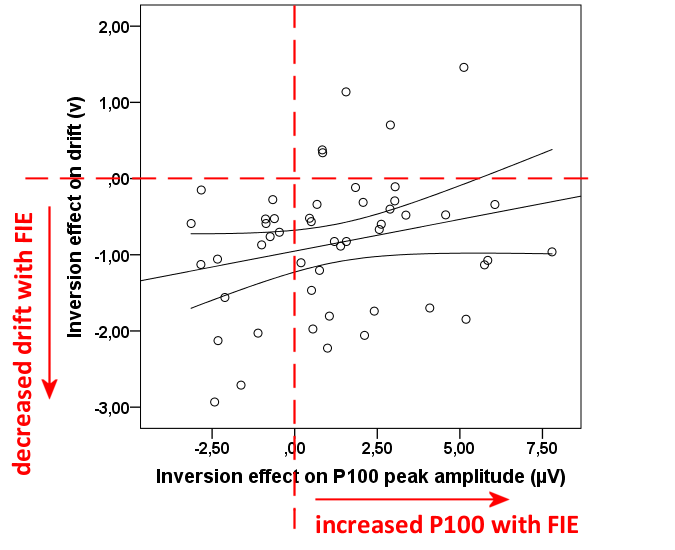
**Supplementary Figure 5.** Illustration of the positive link between FIE on *v* and on P100 peak amplitude.

*
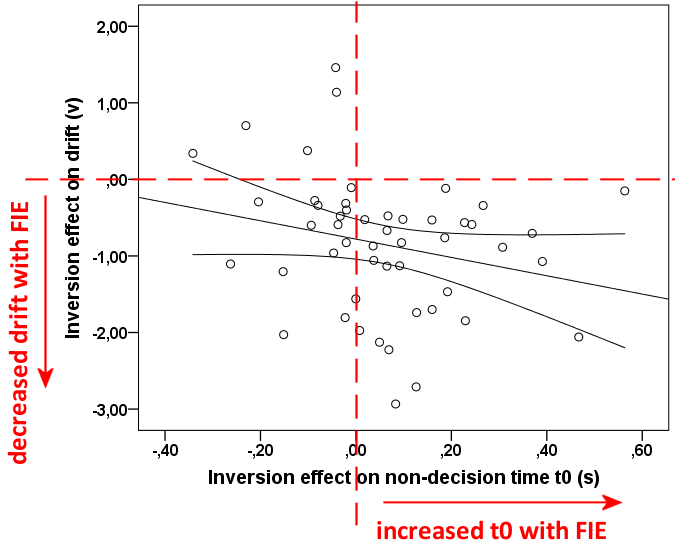
*

**Supplementary Figure 6.** Illustration of the negative link between FIE on *t*_0_ and on drift. FIE on drift decreases with increasing FIE on *t*_0_.

|  |  | |  | Pearson | | | | |  | Spearman | | | | |
| --- | --- | --- | --- | --- | --- | --- | --- | --- | --- | --- | --- | --- | --- | --- |
|  |  | |  | r | *p* | VS-MPR | Lower 95% CI | Upper 95% CI |  | rho | *p* | VS-MPR | Lower 95% CI | Upper 95% CI |
| *v* | - | *t*_0_ | | -0.242 | 0.091 | 1.691 | -0.487 | 0.039 |  | -0.287* | 0.044 | 2.688 | -0.523 | -0.009 |
| *v* | - | P100_lat | | -0.027 | 0.854 | 1.000 | -0.303 | 0.254 |  | 0.043 | 0.766 | 1.000 | -0.238 | 0.318 |
| *v* | - | N170_lat | | 0.099 | 0.495 | 1.000 | -0.185 | 0.367 |  | 0.119 | 0.412 | 1.000 | -0.165 | 0.384 |
| *v* | - | P250_lat | | -0.038 | 0.792 | 1.000 | -0.313 | 0.243 |  | -0.034 | 0.817 | 1.000 | -0.309 | 0.247 |
| *t*_0_ | - | P100_lat | | 0.106 | 0.462 | 1.000 | -0.177 | 0.374 |  | 0.152 | 0.293 | 1.023 | -0.132 | 0.413 |
| *t*_0_ | - | N170_lat | | -0.023 | 0.874 | 1.000 | -0.299 | 0.257 |  | -0.036 | 0.802 | 1.000 | -0.312 | 0.244 |
| *t*_0_ | - | P250_lat | | -0.010 | 0.943 | 1.000 | -0.288 | 0.269 |  | -0.001 | 0.993 | 1.000 | -0.280 | 0.277 |
| P100_lat | - | N170_lat | | 0.016 | 0.912 | 1.000 | -0.263 | 0.293 |  | 0.001 | 0.994 | 1.000 | -0.277 | 0.279 |
| P100_lat | - | P250_lat | | 0.184 | 0.201 | 1.140 | -0.100 | 0.440 |  | 0.211 | 0.142 | 1.327 | -0.072 | 0.462 |
| N170_lat | - | P250_lat | | 0.298* | 0.036 | 3.092 | 0.021 | 0.532 |  | 0.290* | 0.041 | 2.816 | 0.013 | 0.526 |

**Supplementary Table 3.** Correlation matrices on the inversion effect for DDM parameters and ERPs peak latency. **p*< 0.05, ** *p* < 0.01, *** *p* < 0.001

|  |  | |  | Pearson | | | | |  | Spearman | | | | |
| --- | --- | --- | --- | --- | --- | --- | --- | --- | --- | --- | --- | --- | --- | --- |
|  |  | |  | r | *p* | VS-MPR | Lower 95% CI | Upper 95% CI |  | rho | *p* | VS-MPR | Lower 95% CI | Upper 95% CI |
| *v* | - | *t*_0_ | | -0.267 | 0.061 | 2.164 | -0.508 | 0.012 |  | -0.348* | 0.014 | 6.307 | -0.571 | -0.078 |
| *v* | - | P100_amp | | 0.171 | 0.234 | 1.082 | -0.112 | 0.429 |  | 0.038 | 0.795 | 1.000 | -0.243 | 0.313 |
| *v* | - | N170_amp | | -0.268 | 0.060 | 2.185 | -0.509 | 0.011 |  | -0.278 | 0.050 | 2.444 | -0.517 | 0.000 |
| *v* | - | P250_amp | | -0.158 | 0.272 | 1.039 | -0.418 | 0.126 |  | -0.155 | 0.280 | 1.032 | -0.416 | 0.129 |
| *t*_0_ | - | P100_amp | | -0.029 | 0.844 | 1.000 | -0.304 | 0.252 |  | -0.047 | 0.743 | 1.000 | -0.322 | 0.234 |
| *t*_0_ | - | N170_amp | | 0.189 | 0.188 | 1.171 | -0.094 | 0.444 |  | 0.183 | 0.204 | 1.134 | -0.101 | 0.439 |
| *t*_0_ | - | P250_amp | | 0.120 | 0.405 | 1.000 | -0.163 | 0.386 |  | 0.125 | 0.387 | 1.000 | -0.159 | 0.389 |
| P100_amp | - | N170_amp | | -0.102 | 0.481 | 1.000 | -0.370 | 0.182 |  | -0.119 | 0.411 | 1.000 | -0.384 | 0.165 |
| P100_amp | - | P250_amp | | 0.343* | 0.015 | 5.949 | 0.072 | 0.567 |  | 0.462*** | < 0.001 | 62.383 | 0.211 | 0.656 |
| N170_amp | - | P250_amp | | 0.155 | 0.282 | 1.031 | -0.129 | 0.416 |  | 0.066 | 0.650 | 1.000 | -0.217 | 0.338 |

**Supplementary Table 4.** Correlation matrices on the stimulus type effect for DDM parameters and ERPs peak amplitude. **p*< 0.05, ** *p* < 0.01, *** *p* < 0.001

|  |  | |  | Pearson | | | | |  | Spearman | | | | |
| --- | --- | --- | --- | --- | --- | --- | --- | --- | --- | --- | --- | --- | --- | --- |
|  |  | |  | r | *p* | VS-MPR | Lower 95% CI | Upper 95% CI |  | rho | *p* | VS-MPR | Lower 95% CI | Upper 95% CI |
| *v* | - | *t*_0_ | | -0.267 | 0.061 | 2.164 | -0.508 | 0.012 |  | -0.348* | 0.014 | 6.307 | -0.571 | -0.078 |
| *v* | - | P100_lat | | 0.165 | 0.252 | 1.059 | -0.119 | 0.424 |  | 0.135 | 0.351 | 1.001 | -0.149 | 0.398 |
| *v* | - | N170_lat | | 0.010 | 0.944 | 1.000 | -0.269 | 0.288 |  | -0.023 | 0.875 | 1.000 | -0.299 | 0.257 |
| *v* | - | P250_lat | | 0.146 | 0.310 | 1.013 | -0.138 | 0.408 |  | 0.164 | 0.255 | 1.055 | -0.120 | 0.423 |
| *t*_0_ | - | P100_lat | | -0.048 | 0.740 | 1.000 | -0.322 | 0.233 |  | -0.096 | 0.507 | 1.000 | -0.365 | 0.187 |
| *t*_0_ | - | N170_lat | | -0.036 | 0.805 | 1.000 | -0.311 | 0.245 |  | -0.064 | 0.658 | 1.000 | -0.336 | 0.218 |
| *t*_0_ | - | P250_lat | | -0.217 | 0.130 | 1.386 | -0.467 | 0.065 |  | -0.241 | 0.091 | 1.683 | -0.487 | 0.040 |
| P100_lat | - | N170_lat | | 0.028 | 0.846 | 1.000 | -0.252 | 0.304 |  | 0.039 | 0.789 | 1.000 | -0.242 | 0.314 |
| P100_lat | - | P250_lat | | 0.120 | 0.406 | 1.000 | -0.164 | 0.386 |  | 0.129 | 0.373 | 1.000 | -0.155 | 0.393 |
| N170_lat | - | P250_lat | | 0.572*** | < 0.001 | 2288.679 | 0.349 | 0.733 |  | 0.487*** | < 0.001 | 138.340 | 0.242 | 0.674 |

**Supplementary Table 5.** Correlation matrices on the stimulus type effect for DDM parameters and ERPs peak latency. **p*< 0.05, ** *p* < 0.01, *** *p* < 0.001

# Appendix D

## Response accuracy (Hu) as a function of emotion

For the sake of simplicity, the analyses reported here will examine to what extent the *Stimulus type x Orientation* interaction varied with *Emotion*. We first performed an ANOVA on *Hu* (arcsined values) while introducing *Emotion (sadness, fear, happiness, neutral, and anger)* as an additional within-subject factor. The *Stimulus type* x *Orientation* x *Emotion* interaction that turned out to be significant (*F*(4, 96) = 4.31, *p* = 0.003, η_p_^2^ = 0.152), reflecting the fact that *Emotion* modulated the *Stimulus type* x *Orientation* interaction on *Hu*, as illustrated in *Supplementary Figure 7*. The emotions contributing mainly to the *Stimulus type* x *Orientation* interaction on *Hu* (see Figure 3 in the main text) were anger and neutral, and to a lesser extent sadness, as illustrated by the similarity of their graphical patterns. However, the higher-order interaction accounted for 3 times less variance (η_p_^2^ = 0.152) than the *Stimulus type* x *Orientation* interaction (η_p_^2^ = 0.524), suggesting that the variance in *Hu* introduced by Emotion was relatively less important than for the first-order interaction.


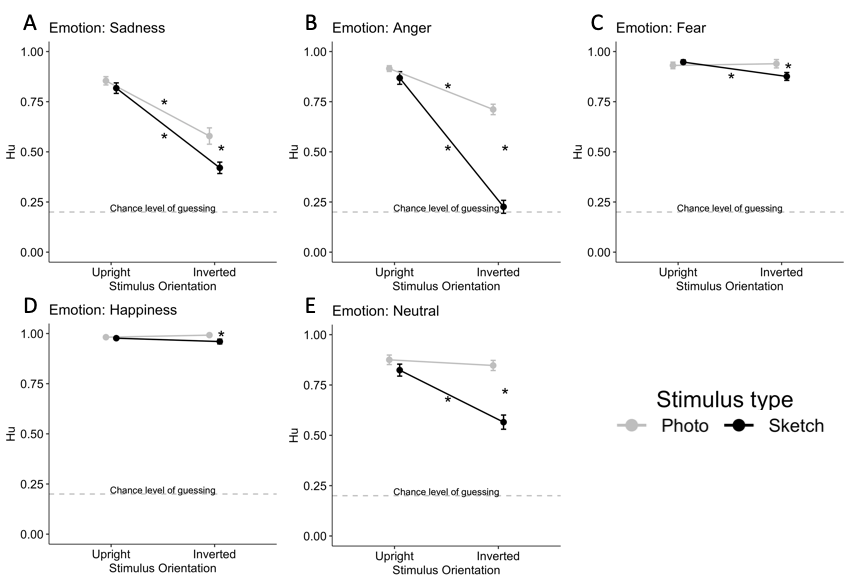


**Supplementary Figure 7.** Illustration of the *Stimulus type* x *Orientation* x *Emotion* interaction on response accuracy (*Hu*). Note that this figure displays the *Hu* values before they were arcsined to run the ANOVAs. Stars indicate significant Bonferroni post-hoc pairwise comparisons, *p* < 0.05.

| **Emotion** | **Source of variance** | ***F* (1, 24)** | ***p* value** | η_p_^2^ |
| --- | --- | --- | --- | --- |
| Sadness | *Stimulus type* | 11.73 | 0.002 | 0.33 |
|  | *Orientation* | 64.81 | <0.0001 | 0.73 |
|  | *Stimulus type* x *Orientation* | 3.62 | 0.069, n.s. | 0.13 |
|  |  |  |  |  |
| Fear | *Stimulus type* | 2.94 | 0.10, n.s. | 0.11 |
|  | *Orientation* | 2.66 | 0.12, n.s. | 0.10 |
|  | *Stimulus type* x *Orientation* | 4.74 | 0.04 | 0.17 |
|  |  |  |  |  |
| Happiness | *Stimulus type* | 8.02 | 0.009 | 0.25 |
|  | *Orientation* | < 1 | 0.95, n.s. | 0.00 |
|  | *Stimulus type* x *Orientation* | 2.01 | 0.17, n.s. | 0.08 |
|  |  |  |  |  |
| Neutral | *Stimulus type* | 42.08 | < 0.0001 | 0.64 |
|  | *Orientation* | 14.41 | 0.001 | 0.38 |
|  | *Stimulus type* x *Orientation* | 16.33 | < 0.001 | 0.41 |
|  |  |  |  |  |
| Anger | *Stimulus type* | 45.33 | < 0.0001 | 0.65 |
|  | *Orientation* | 153.88 | < 0.0001 | 0.87 |
|  | *Stimulus type* x *Orientation* | 31.43 | < 0.0001 | 0.57 |

**Supplementary Table 6.** Results of separate ANOVAs per *Emotion*, on *Hu* (arcsined values).

##

## Correct answer RTs as a function of emotion

We also performed an ANOVA on RTs for correct responses, with *Emotion* as an additional within-subject factor. The *Stimulus type x Orientation x Emotion* interaction turned out to be significant (*F*(4, 96) = 3.51, *p* = 0.01, η_p_^2^ = 0.13), reflecting the fact that *Emotion* modulated the *Stimulus type x Orientation* interaction on correct answer RTs, as illustrated in *Supplementary Figure 8*. However, the higher-order interaction accounted for 2 times less variance (η_p_^2^ = 0.128) than the *Stimulus type x Orientation* interaction (η_p_^2^ = 0.248), suggesting that the variance in RT introduced by *Emotion* was relatively less important than for the first-order interaction. Noteworthy, the RT data show that there was no speed-accuracy tradeoff as illustrated by the data of inverted sketch angry and neutral expressions that show low *Hu*, in spite of high RT values.


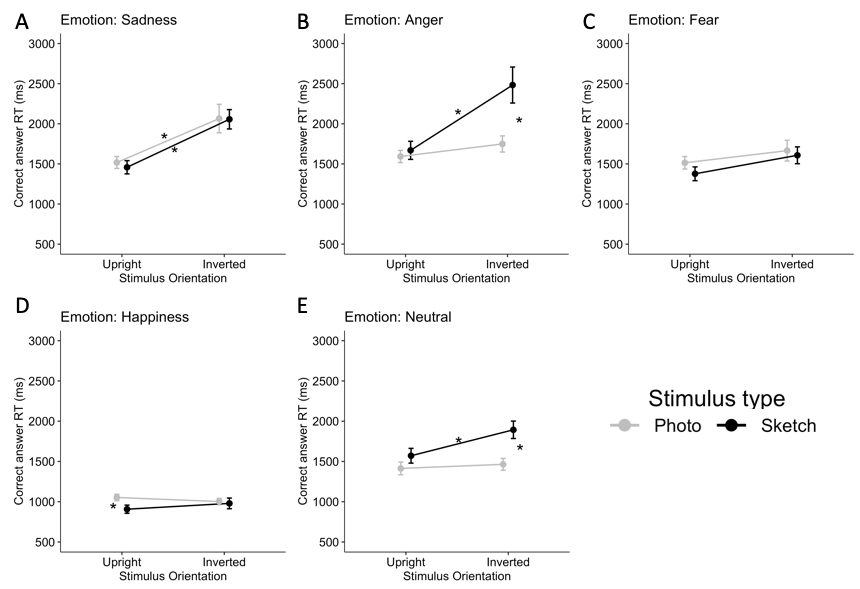


**Supplementary Figure 8.** Illustration of the *Stimulus type* x *Orientation* x *Emotion* interaction on correct answer RTs. Stars indicate significant Bonferroni post-hoc pairwise comparisons, *p* < 0.05.

| **Emotion** | **Source of variance** | ***F* (1, 24)** | ***p* value** | η_p_^2^ |
| --- | --- | --- | --- | --- |
| Sadness | *Stimulus type* | 0.12 | 0.74, n.s. | 0.01 |
|  | *Orientation* | 45.95 | < 0.0001 | 0.66 |
|  | *Stimulus type* x *Orientation* | 0.06 | 0.80, n.s. | 0.00 |
|  |  |  |  |  |
| Fear | *Stimulus type* | 2.07 | 0.16, n.s. | 0.08 |
|  | *Orientation* | 6.63 | 0.02 | 0.22 |
|  | *Stimulus type* x *Orientation* | 0.77 | 0.39, n.s. | 0.03 |
|  |  |  |  |  |
| Happiness | *Stimulus type* | 5.11 | 0.03 | 0.18 |
|  | *Orientation* | 0.09 | 0.76, n.s. | 0.00 |
|  | *Stimulus type* x *Orientation* | 4.08 | 0.06, n.s. | 0.15 |
|  |  |  |  |  |
| Neutral | *Stimulus type* | 15.64 | < 0.001 | 0.40 |
|  | *Orientation* | 10.93 | 0.003 | 0.31 |
|  | *Stimulus type* x *Orientation* | 8.93 | 0.006 | 0.27 |
|  |  |  |  |  |
| Anger | *Stimulus type* | 11.87 | 0.002 | 0.33 |
|  | *Orientation* | 21.29 | 0.0001 | 0.47 |
|  | *Stimulus type* x *Orientation* | 8.72 | 0.007 | 0.27 |

**Supplementary Table 7.** Results of separate ANOVAs per *Emotion*, on correct answer RTs.

# References

Voss, A., Nagler, M., & Lerche, V. (2013). Diffusion models in experimental psychology. *Experimental Psychology*, *60*(6), 385-402.

Voss, A., Rothermund, K., Gast, A., & Wentura, D. (2013). Cognitive processes in associative and categorical priming: A diffusion model analysis. *Journal of Experimental Psychology: General*, *142*(2), 536-559.

Voss, A., & Voss, J. (2007). Fast-dm: A free program for efficient diffusion model analysis. *Behavior Research Methods*, *39*(4), 767-775.
